# 

**Published:** 1888-12-29

**Authors:** 


### CONTENTS

**"Ring Out the Old"** - - - - - 193

**A Plea for Our Voluntary Hospitals** - - - - 194

**A Christmas Carol in a Minor Key.**—The Gospel of Selfishness—The Thread is Broken—A Chance Step—Among the Toys—A Plea for the Dolls—Best of All—Sick Children—Fact versus Fancy—Why one Avoids the Hospitals—At a Children's Hospital—The Crown of All—In the East—Music and Dancing—Fun for All—A Change of Temper—Here and There and Everywhere—What Would the Subscribers Say?—A Plea for Amusement—The Philosophy of Christmas—Where the Money Comes From—Accomplished Nurses—The Fascination of Hospitals—Are Hospitals Extravagant?—The Result of Rash Accusations—The Meaning of Pain—Who are the Fittest?—What Hospitals Teach—The Practical Lesson—Good-bye to Selfishness - - - 195

**Notes and News** - - - - 200

**Annotations.**—"Let Him Die."—Hospitals at Home &c. - 201

**Everybody's Page** - - - - 202

**The Philanthropist's Vade Mecum** - - - 203

**EXTRA SUPPLEMENT.—THE NURSING MIRROR**

**En Passant.**—Riches and Poverty—Short Items—Lectures to Infirmary Nurses—Soirée at the London—Nurses at Alexandria—Hospital Extravage—Philanthropic Sweating xlix

**Letters from a Probationer.**—VI.—Matron at Home - l

**Notes from Australia** - - - l

**The National Pension Fund for Nurses** - - li

**Everybody's Opinion.**—Rest and Exercise - - lii

**The Nurses' Bookshelf.**—"Laying Out the Dead" - lii

**Lectures** - - - - li

**Vacancies—Notes and Queries** - - - - lii

